# Do learners implement what they learn? Commitment-to-change following an interprofessional palliative care course

**DOI:** 10.1177/02692163221081329

**Published:** 2022-03-08

**Authors:** José Pereira, Lynn Meadows, Dragan Kljujic, Tina Strudsholm

**Affiliations:** 1 Pallium Canada, Ottawa, Canada (Non-profit Foundation); 2 Division of Palliative Care, Department of Family Medicine, McMaster University, Hamilton, Canada; 3 Institute for Culture and Society (ICS), University of Navara, Spain; 4 Department of Community Health Sciences, Cumming School of Medicine, University of Calgary, Calgary, AB, Canada; 5 Database Manager and Analyst; 6 School of Health Sciences, University of Northern British Columbia, Prince George, BC, Canada

**Keywords:** Palliative care, continuing education, interdisciplinary studies, evaluation, interprofessional, commitment to change

## Abstract

**Background:**

Palliative care educators should incorporate strategies that enhance application into practice by learners. Commitment-to-change is an approach to reinforce learning and encourage application into practice; immediately post-course learners commit to making changes in their practices as a result of participating in the course (“statements”) and then several weeks or months later are prompted to reflect on their commitments (“reflections”).

**Aim:**

Explore if and how learners implemented into practice what they learned in a palliative care course, using commitment-to-change reflections.

**Design:**

Secondary analysis of post-course commitment statements and 4-months post-course commitment reflections submitted online by learners who participated in Pallium Canada’s interprofessional, 2-day, Learning Essential Approaches to Palliative Care (LEAP) Core courses.

**Setting/participants:**

Primary care providers from across Canada and different profession who attended LEAP Core courses from 1 April 2015 to 31 March 2017.

**Results:**

About 1063 of 4636 learners (22.9%) who participated in the 244 courses delivered during the study period submitted a total of 4250 reflections 4 months post-course. Of these commitments, 3081 (72.5%) were implemented. The most common implemented commitments related to initiating palliative care early across diseases, pain and symptom management, use of clinical instruments, advance care planning, and interprofessional collaboration. Impact extended to patients, services, and colleagues. Barriers to implementation into practice included lack of time, and system-level factors such as lack of support by managers and untrained colleagues.

**Conclusions:**

Examples of benefits to patients, families, services, colleagues, and themselves were described as a result of participating in the courses.

**What is already known in this area**

- Commitment-to-change is a multipurpose approach to reinforce learning and to evaluate the impact of an education program.
- In commitment-to-change, learners commit immediately post-course to making changes to their practices (“statements”) as a result of participating in an educational program. Several weeks or months later they are prompted to reflect on their commitments (“reflections”), self-report on the extent to which they implemented them, and describe the impact of the changes.

**What this research adds**

- This study provides evidence that primary care professionals, including physicians and nurses, who participated in a 2-day palliative care course did, to varying degrees, apply into practice what they learned during the course.
- Learners who submitted reflections 4 months after the course self-reported that they had implemented three quarters of commitments they made.
- Learners self-reported impact on patients and families, services, colleagues, and across areas such as initiating palliative care earlier, advance care planning and goals of care discussions, opioid use, symptom management and interprofessional collaboration.

**Implications for clinical practice**

- Commitment-to-change offers a useful approach for palliative care educators.
- Palliative care continuing professional learning programs, with appropriate designs, do impact patients, health care providers and the health care system positively.

## Introduction

Palliative care education remains a key strategy to address the needs of patients with palliative care needs.^1,2–4^ Curriculum gaps and learning needs related to palliative care have been identified across the learning cycle of health care professionals, from undergraduate and postgraduate training to continuing education for clinicians in practice.^5–7^ There is emerging evidence that courses of varying length do address some of these gaps.^8^

Several strategies that enhance the effectiveness of educational programs have been described. These include interactivity, problem- or case-based learning, follow-up with reminders, feedback, and peer discussions, and forced functions such as prompts in electronic medical records.^9–11^ Moreover, educators are increasingly expected to evaluate impact on patients and the health care system in addition to constructs such as the learner experience and changes in knowledge, attitudes, self-efficacy and skills.^12–14^

Commitment to change is a widely used approach in medical continuing education to reinforce learning and to encourage learners to apply what they learn in their daily practice.^15–17^ However, it is seldom reported in palliative care. It has been used to provide learner feedback, reinforce behavioral change, and facilitate reflective practice.^18–21^ Increasingly it is also used to evaluate the impact of education interventions and programs.^15,18,22–24^ Several studies, including randomized trials, show that clinicians who express a commitment to change post-intervention and those who reflect on them at a later time are more likely to change their behavior in practice; thereby increasing the likelihood of follow-through and implementation.^25–29^

There are many variations in how the commitment-to-change approach is applied.^15^ Some use a post-only design, where learners identify areas for change and commitments immediately after the course. Others use a post/follow-up design where learners submit post-course commitments and then receive follow-up reminders weeks or months later with requests to reflect on them and the extent to which they were implemented into practice.^30,31^ The post/follow-up approach is increasingly preferred.^18,23,24,28,30–32^ Procedures for collecting commitment-to-change statements vary. Some use an open-ended approach in which learners submit their commitment statements in free text, while others provide lists of potential change ideas that learners select from; open-ended statements may be more effective.^24^ A scale is sometimes added to each commitment for learners to indicate their level of commitment to enhance the reflective nature of the exercise and to help prioritize.

Approaches for undertaking the follow-up also vary. These include the time frame between making the statements and follow-up reflections, types of scales used to assess implementation, inclusion of questions soliciting feedback on barriers encountered, additional changes made, or new commitments since completing the original statements. Statements should ideally be linked to the course’s learning objectives.^33^ In-depth explanations of the theoretical and practical foundations, and ethical implications underlying commitment-to-change are described in the literature.^32,34^

The commitment-to-change approach has generally not been reported in palliative care. Pallium Canada, a non-profit organization that builds primary-level palliative care capacity through interprofessional education courseware called Learning Essential Approaches to Palliative Care (LEAP), has been using the commitment-to-change approach for over a decade.^4^ The LEAP program consists of a suite of interprofessional courses, each one targeting a different care setting or disease group. The main goal of the LEAP courses is to provide health care providers with the core competencies to provide a palliative care approach.^35,36^ These are core palliative care skills, provided by non-palliative care specialists, and include identifying patients with palliative care needs early, undertaking essential conversations such as advance care planning and goals of care discussions, decision-making, assessing and managing pain and other symptoms, and addressing psychosocial and spiritual needs across the illness trajectory.

## Aims

This study sought to explore, using commitment-to-change statements and reflections, whether learners who have participated in LEAP Core courses implement what they learned into practice. The study also sought to understand the nature of the changes they implemented and examples of the impact.

## Methods

### Overall design

A secondary analysis of existing data that had previously been collected was undertaken. This data consisted of immediately post-course commitment-to-change statements and 4-months post-course commitment-to-change reflections.

### Setting and intervention

The course version reported in this study is the LEAP Core classroom version. LEAP Core targets primary care providers, including family medicine practices and home care agencies. It consists of 13 modules delivered over 14 hours (usually delivered over two consecutive days) and incorporates learning methods such as small group case-based learning to ensure interactivity and interprofessionality. Courses are limited to a maximum of 30 learners per session and are facilitated by an interprofessional team of two or three palliative care physicians and nurses. Although the courses largely target physicians, nurses, pharmacists and social workers, other professions are also welcomed. In-depth descriptions of the course’s goals and learning objectives, design, and curriculum development have been published previously.^4,37,38^

Learners are asked at the end of the last session of the course to identify three to four things that they will do differently because of participating in the course. These are referred to as the post-course commitment statements. They enter these, using free text, online into Pallium Canada’s learning management system (LMS). To avoid cueing, no lists or drop-down menus of potential commitments are provided.^33^

Four months after completing the course, each learner who submitted post-course commitment statements is automatically sent a personalized email by the LMS. The message lists the three to four commitments the learner submitted immediately post course. For each commitment statement, they are asked to indicate, using one of three response options, whether they have implemented the commitment into practice; (a) “I have had no opportunity to implement the change”; (b) “I had opportunities but have not implemented the change”; and (c) “I have implemented the change.” For each implemented statement they are asked to provide an example of the impact of implementing the change. For commitments that they were not able to implement, they are asked to describe barriers that prevented them from implementing them. A 4 month period was chosen to allow sufficient time and opportunities to implement the changes.^33^

Submission of the post-course commitment statements and the 4-months commitment reflections are voluntary. However, for physicians who wish to apply to their professional bodies for continuing medical education credits, completion of the statements and reflections is mandatory.

### Population and sample

We studied the post-course commitment statements and 4-months commitment reflections submitted by LEAP learners across professions for all LEAP Core courses delivered over a 2-year period; 1 April 2015 to 30 March 2017. This period was chosen as it represented the first 2 years that this data was collected using the new LMS system.

### Data collection and management

All LEAP courses and learners are registered online in Pallium Canada’s customized *Moodle*-based LMS. The LMS is used to submit all the course-related surveys and questionnaires, including pre- and post-course knowledge, attitudes and comfort questionnaires and the commitment-to-change statements and reflections.

All data were downloaded from the LMS databases into a Microsoft Excel™ 2016 spreadsheet and checked for quality, cleaned, and de-identified by a database manager. The data were then shared with the qualitative analytic team through a secure data base link. Data organization and coding was aided by using the NVivo 12 Pro software program.

### Analysis

A traditional iterative qualitative analysis approach that involved coding, followed by identification of emerging themes and illustrative quotes was used.^39–41^ Two researchers (LM, TS) did the initial coding independently and then met to confirm codes and themes through consensus. A third researcher (JP) provided additional context and verification. Rigor was maintained through independent coding reviews, interdisciplinary team discussions and bracketing.^42–44^ This process also included identifying topics that although not mentioned very often, had the potential for significant impact on patients (referred to in this paper as “saliency” analysis).

Although in qualitative data analysis the goal is to identify and describe the nature of the data, not to quantify it, we also undertook a quantitative, enumerative analysis of the statements and themes to provide an indication of frequency and prevalence of common commitments made and reported by respondents.^45^

### Ethics

The study was reviewed and approved by the Conjoint Health Research Ethics Board (CHREB) of the University of Calgary (REB 17-0429).

## Results

### Response and implementation rates

A total of 244 courses were delivered during the study period with a total of 4636 participants (See Table 1). Professionals from various professions participated in the courses. Nurses (including registered nurses, registered practical nurses and nurse practitioners) made up the largest proportion (2990; 65%), followed by physicians (878; 19%). Pharmacists and social workers made up 4.9% of all learners. The group “Others” included physiotherapists, occupational therapists, counsellors, and administrators (541; 12%).

**Table 1.** LEAP Core Commitment-to-Change statements and reflections: Response rates immediately post-course and 4-months post-course, and implementation rates 4-months post course.

	All learners	Physicians	Nurses	Pharms	Social workers	All others
Number of learners in courses (%)	4636	878 (19%)	2990 (65%)	100 (2%)	127 (3%)	541 (12%)
Statements immediately post-course^a^						
Number of learners who responded (% of all learners)	2574	559	1672	66	64	213
Response rate^a^	56%	64%	56%	66%	50%	40%
Total number of statements^b^	10288	2231	6685	264	256	852
Reflections 4-months post course						
Number of learners who responded to 4-month post reflection survey	1063	226	664	24	33	116
Response rate relative to number of learners who posted CTC statements immediately post-course^c^	42%	40%	407%	36%	52%	55%
Response rate relative to number of learners enrolled in courses^d^	23%	26%	22%	24%	26%	21%
Total number of statements reflected on^e^	4250	904	2654	96	132	464

Pharms: pharmacists.

a Response rate: [Number of learners who responded divided by number of learners who enrolled in courses] × 100.

b Learners commit, immediately after each course, to change three to four things in their practices as a result of participating in the course (commitment statements). Then 4 months post-course they are shown their original commitment statements and asked to reflect on them (reflection), and to indicate whether they implemented them (“Yes”, “No,” or “No because of no opportunity”). They are asked to provide examples of the impact, or barriers if not implemented.

c Response rate: [Number of learners who responded to Reflection survey 4 months post-course divided by number of learners who completed Statements immediately post-course] × 100.

d Response rate: [Number of learners who responded to 4 months post-course Reflection divided by number of learners who enrolled in courses] × 100.

e Total number of commitment statements made immediately post-course that were reflected on 4 months post-course.

Table 1 also presents the response rates to the post-course statements and the 4-months reflections. A total of 2574 learners (56% of all the learners enrolled in the courses) submitted post-course commitment statements. A total of 10,288 statements were submitted post-course.

For the 4-months post-course reflections, 1063 learners submitted reflections on their initial commitment statements (See Table 2). This represented 42% of all learners who had submitted post-course statements and 23% of all learners who enrolled in the course. A total of 4250 reflections were submitted. Of those, 3081 (73%) were reported by learners as implemented. A total of 339 (8%) commitments were not implemented despite opportunities to do so. The extent to which learners implemented their commitments varied across professions.

**Table 2.** Implementation rates of Commitment-to-Change statements 4-months post-course of LEAP Core course participants.

	Total	Physicians	Nurses	Pharms	Social workers	All others
Number of learners who provided 4-months post course reflections^a^	1063	226	664	24	33	116
Total number of commitment statements reflected on 4-months post course^a^	4250	904	2654	96	132	464
Number of statements **implemented** (%)^b^	3081 (73%)	668 (74%)	1946 (73%)	69 (72%)	80 (61%)	318 (69%)
Number of statements for which **no opportunities to implement** presented (%)	830 (20%)	169 (19%)	500 (19%)	22 (23%)	38 (29%)	101 (22%)
Number of statements **not implemented**	339 (8%)	67 (7%)	208 (8%)	5 (5%)	14 (11%)	45 (10%)

Pharms: pharmacists.

a Learners commit, immediately after each course, to change three to four things in their practices as a result of participating in the course (commitment statements). Then 4 months post-course they are shown their original commitment statements and asked to reflect on them (reflection), and to indicate whether they implemented them (“Yes”, “No,” or “No because of no opportunity”). They are asked to provide examples of the impact, or barriers if not implemented.

b Number of commitments made post-course that had been implemented as reported by learners in the 4-month post-course reflection (% refers to proportion of total number of commitments submitted 4 months post-course).

### Post-course commitment statements

Several themes emerged from the post-course commitment statements and the 4-months post-course reflections. The most frequently occurring themes are listed in Table 3. These included initiating palliative care early across disease groups, opioid use and managing symptoms, use of clinical instruments, advance care planning, and interprofessional collaboration.

**Table 3.** LEAP Core Commitments-to-Change Post-course and 4 months post-course: Most frequently occurring statements by themes.

Themes	Number of statements per theme (%)*	
	Post-course	4 months post-course
Number of commitment statements and reflections	10,288	1063
Early initiation of palliative care and principles of palliative care	1259	604
Opioids and other medications	1129	518
Increased use of screening and assessment clinical tools	733	134
Pain management	273	85
Advance care planning	273	74
Symptom management (other than pain)	219	57
Hydration or nutrition issues	122	38
Palliative Sedation	103	44
Grief and bereavement care	97	24

These numbers are generated through coding techniques that provide a relative indication of the frequency of occurrence, rather than an exact number. Some participants mentioned more than one theme in a single commitment statement and in other cases themes overlapped.

* Percentage relative to the total number of statements/reflections submitted.

Table 4 lists the top five most occurring themes with illustrative quotes. For the theme related to initiating palliative care earlier, the statements focused largely on identifying patients with palliative care needs earlier, initiating a palliative care approach earlier and helping patients and families to understand the palliative approach and that it was not limited to end-of-life. This theme highlighted principles of palliative care; that is not restricted to end-of-life (last days or weeks of life). In the theme related to opioid use and other medications, a broad range of medications were covered. Learners indicated increased confidence using palliative care-related medications appropriately. The commitments related to pain and symptom management reflected a broad range of pain and symptom assessment and management approaches. In the advance care planning (ACP) theme, statements focused largely on the elements and processes of ACP and better use of ACP resources.

**Table 4.** LEAP Core Commitments to Change (CTC) immediately post-course: Top five most occurring themes with illustrative quotes and comments (all profession groups).

Emerging themes	Sample quotes
Early initiation of palliative care	*I will be more proactive and open in my conversations with patients in accessing the Palliative care service early in a patient’s disease process (Nurse)*
Opioids and other medications	*I will make recommendations for a laxative regimen for every patient starting opioid medications. (Pharmacist)*
	*I will be more aware of the signs of opioid toxicity and look to hydrate the patient and either reduce the dose or rotate the opiod. (Physician)*
Increased use of screening and assessment clinical tools	*Consistent use of screening tools (ESAS, PPS, FICA etc.). (Social worker)*
	*I will use the screening tools - ESAS and PPS more often in my assessments, I will also use them at the beginning of the palliative care process. (Physician)*
Pain and symptom management	*“. . . to continue to practice the calculations in rotation of pain medication.” (Physician)*
	*Monitor pain management once implemented - monitor efficacy, delirium, neurotoxicity, etc. (Pharmacist)*
	*Consider opioids as only one part of the total pain management plan (Physician)*
Advance care planning (ACP)	*I will ensure that my palliative care clients know why advance care planning is necessary. . .. (Nurse)*
	*Implement advance care planning into regular practice (Physician)*
	*To be more aware of resources available - especially advance care planning to be able to share with patients that ask as well as direct family members to make their decisions. (Pharmacist)*

Overall, the post-course commitment statements signaled that study participants, across profession groups, demonstrated application of a palliative care approach. This included, among others. better symptom management and the regular use of symptom and needs screening tools. Learners reported sharing their newly acquired knowledge and skills with nursing colleagues. Some nurses described increased ability to engage physicians regarding patient care plans. Some participants mentioned providing more holistic care.

### 4-months commitment reflections

The themes that emerged from the 4-months reflections and illustrative quotes are summarized in Table 5. Practice changes related to the theme “early initiation of palliative care” included initiating conversations about palliative care earlier and being more proactive in beginning a palliative care approach for cancer as well as non-cancer populations. Commitments implemented related to “opioids and medications” manifested as better opioid and medication dosing and titration, and the use of different modalities for pain and symptom control. Nurses described identifying patient discomfort better and responding to these quicker, including using non-pharmacological methods. A broader use of different clinical tools such as the Surprise Question, the Edmonton Symptom Assessment Scale (ESAS-V2) and the Palliative Performance Scale (PPS-V2) was described. In some cases, participants spread the use of these tools throughout their services. In the theme “pain and symptom management”, practice change led to observed improved symptom management, both physical and psychological. More appropriate management of hydration and nutrition was described in this theme.

**Table 5.** LEAP Core Commitment-to-Change reflections 4 months post-course: Examples of impact as described by learners across the top seven most occurring themes with illustrative quotes.

Themes	Sample quotes
Early initiation of palliative care	*Through the pallium course I have realized the benefits of early referral to the palliative team for counselling, support, and many other aspects of palliative care that I did not realize before. This is a big benefit to both the patient and their families. (Physician)*
	*Palliative care is not just for cancer patients who are dying. I have better knowledge now to screen patients who could benefit from palliative care (Physician)*
	*I now make a conscious effort to introduce the idea of palliative care as a symptom management modality when an illness is incurable rather than just an end-of-life toolkit. (Physician)*
Opioids and medications	*I recently had a patient in community setting being switched from one opioid to another and it required consultation with physician to correct the dosage. It had a huge impact on patent care because patient would have been significantly under dosed and pain control would have suffered. (Pharmacist)*
	*When doing physician rounds at the manor I have been able to advocate on behalf of residents who need additional or a change in pain medication. (Nurse)*
Increased use of screening and assessment clinical tools	*In my hospital role, as a team member in Medically Complex Care, I have been able to ensure the discussion at patient rounds includes the use of standardized tools to more appropriately engage our available palliative care team. (Nurse)*
	*I have used the Palliative Performance Scale and the Edmonton Symptom Assessment System. The first helps to communicate with other Health Care providers. . . ..(Physician)*
Advocacy	*We often work together with patients’ families as well as physicians when it comes to covering costs of some palliative medications. If the prescribing physician does not have the PCFA license, we attempt to contact that physician to see if there is an alternate physician caring for the patient who does possess this license. . .. (Pharmacist)*
Pain and symptom management	*Adjusting medications based on symptoms and addressing constipation issues early on with multiple therapies such as PEG (Pharmacist)*
	*I have been more compliant with ESAS and with reviewing/implementing the symptom management guidelines through an app on my phone. (Nurse)*
Grief and bereavement care	*“I created an education session which I have provided twice on evidenced based Grief and Bereavement Care. The tools are now available. and are being implemented with the support of Management and our National Educator. I have used the tools myself and have provided several bereavement visits since the education session in November.” (Nurse)*
	*I have taken additional training on palliative care support and grief and loss. This has helped me understand my bias with palliative care. I have weekly debriefings with my co-worker and supervisor. (Social Worker)*
Advance care planning	*I have been able to discuss advance care planning with all my patients who a frail and elderly, for whom I provide home visits. Also, with COPD, HF and chronic conditions [Physician]*
	*The knowledge I learned has helped me to have effective conversations with clients and their caregivers. For example, I had a client who was not accepting of his situation. By making little steps with the communication strategies I was able to assist him in completing POA documents and starting conversations with his family about is wishes at EOL. [Nurse]*
	*I have set up reminders in EMR and discussed at patient subsequent visits. [Physician]*
	*I spoke with one family on admission where their Mother had quite advanced Parkinsons and the family had not discussed with the resident her view on DNR status. The family seemed to have conflicting view on this but as the resident was cognitive I encouraged them to speak with their Mother. Several days later the resident came to see me to put a DNR in place. She thanked me for helping her open up the discussion with her family as she knew they did not agree. [Physician]*
	*In my personal life, I have had opportunity to encourage ACP for my friend recently diagnosed with cancer and she has completed all aspects. [Nurse]*

The theme of “grief and bereavement care” was most often mentioned by nurses. This included comments on dealing better with their own grief at losing a patient and the grief of family members. Learners across professions described the impact of practice change related to ACP; initiating ACP discussions more often and earlier across disease groups, using resources more often, sharing resources with patients and families, and promoting ACP amongst colleagues. The course prompted some learners to undertaken ACP themselves and to promote it amongst family members and friends.

“Advocacy” emerged in the 4-months reflections more predominantly than in the post-course statements, especially amongst nurses, pharmacists, and social workers. It included advocacy for patient needs, resources, and system changes. Examples of initiating quality improvement initiatives were also provided. Some learners, for example, initiated projects to integrate reminders of ACP in their practice’s electronic medical records.

A comparison across the profession groups showed similarities across the themes (see Table 6). Nurses’ comments did however show a determination to be an active and informed voice in providing a palliative care approach. Physicians’ reflections demonstrated a deeper understanding of early palliative care, more interprofessional collaboration and the increased use of clinical tools. Social workers described increased use of ACP resources, to support their work.

**Table 6.** Most frequently implemented Commitment-to-Change commitments by themes 4-months post-course for different profession groups (not specifically in any order).

Nurses:	Symptom management
	Pain management (other than opioids)
	Grief and bereavement care
	Opioids and other medications
	Hydration and nutrition
	Advance care planning
Physicians:	Advance care planning
	Opioids and other medications
	Pain management (other than opioids)
	Hydration and nutrition
	Symptom management
	Palliative sedation
Pharmacists:	Opioids and other medications
	Symptom management
	Palliative sedation
	Hydration and nutrition

Several themes were identified in the saliency analysis; (a) improved communication and collaboration with other professions, (b) improved palliative sedation, and (c) empowered to challenge the status quo or “old” ways of doing things.

*I almost always have discussions with the nurses and/or physicians when I see hydromorphone and midazolam continuous infusions started. This course has given me the knowledge and confidence to do all of the above. [Pharmacist]*

*The day after this course I was asked to do palliative sedation on a client out of my district. . . . did have an RN present with me to verify doses, meds, frequencies, pump settings, vitals, etc. Also had direct contact with manager and pharmacy . . .. Successful palliative sedation. Family pleased and client very comfortable. [Nurse]*

Several barriers were described by respondents. These included lack of time because of busy clinic schedules, system-level factors, and colleagues or managers who were not acquainted with the palliative care approach. This was the source of frustration and even moral distress for some as they described being blocked from making applying newly acquired knowledge and skills. Some described ongoing efforts to implement commitments.

*Unfortunately, this goal has not been met although this change remains a commitment of mine. I had met with the director of collaborative practice within my organization with the hopes of proposing a number of changes to documentation of patient wishes as well an interdisciplinary education on end-of-life care and advance care planning. [Nurse]*

*I have implemented this change . . .however did learn that my request [referral to a specialist team] was denied when requested early. Due to the overwhelming demand for palliative care, the palliative team did not get involved until PPS was about [30%] or [less]. They did not want to see the Client until all chemo and radiation treatments were complete even if cancer was wide spread and treatment was only to get more time. What I was taught in the course about requesting early is not the reality. [Nurse]*

Commitments that were more frequently not implemented included using opioid continuous infusion pumps, managing opioid neurotoxicity and managing major depression in this patient population. The most common reason was that no patients with these needs presented post-course. Some reported that they were too busy to incorporate clinical tools like symptom screening instruments while others perceived these to be too time-consuming and burdensome for patients and care providers.

## Discussion

### Main findings

This study provides evidence that core palliative care competencies related to providing a palliative care approach acquired during a 2-day palliative care course were implemented by primary care health care professionals from across profession groups who participated in the study, at least up to 4 months following the course. It also provides examples of the impact of implementing the newly acquired competencies on patient care and services. Benefits related to interprofessional collaboration were also described.

Overall, just over half of learners (56%) submitted post-course commitment statements. These post-course completion rates are consistent with rates reported by others in non-palliative care contexts.^15,17,18,21,23–25,46–48^ The response rate at 4 months post-course for the reflections was lower (23%). These lower compliance rates are not surprising as participating in the commitment-to-change statements and reflections was largely not obligatory and health care professionals often have many competing priorities.

For learners who submitted post-course statements and 4-month post-course reflections, almost three quarters (73%) of commitments were self-reported as implemented. The highest rates were amongst physicians and nurses (74% and 73%) and the lowest amongst social workers (61%). These rates are similar to rates reported previously.^17–19,21,23–25,30,46^ Only a small number of commitments (8%) had not been implemented despite opportunities to do so. Adams et al.,^48^ in a course related to managing chronic airway disease, reported similar low rates of non-implementation despite opportunities (6%).

Some of the barriers to implementing commitments in our study were similar to those described by others.^24,47,49^ Rehring et al.,^24^ for example, described barriers across five categories, namely time barriers (competing demands), clinician (hard to change habits, worry about clinical or legal consequences), staff (staff shortages, lack of training and incentives), organizational (lack of support from managers or leaders, costs, computerized aids, organizational priorities), and patient-related barriers (complexity, beliefs and expectations).

For some, the newly acquired knowledge and confidence was the source of frustration and even moral distress when they encountered barriers to implementing their new-found skills in the workplace. This phenomenon has been described elsewhere in palliative care.^50,51^ Traditional power imbalances—where nurses’ opinions on care plans are not as valued as those of physicians’—may lie at the root of some of these experiences, underlining the need for increased interprofessional collaboration.^52^

Dolcourt^33^ noted a differential participation in commitments to change between those professions that have more autonomy (physicians and advanced practice nurses) versus those professions with less (nurses and physician's assistants). In our study the rates of implementation across the professions were quite similar, suggesting the learners in the different professions identified commitments that were mostly within their scope of practices and spheres of influence.

The commitment statements and reflections in our study showed variability in style. This is not surprising as they were free-text responses. This variability does constitute a challenge to researchers when analyzing commitment statements and reflections. Shershneva et al.^15^ have suggested sorting commitment reflections into five categories, namely practice changes, additional learning activities, attitude changes, confirmation of current practice; and new commitments or changes. Overton and MacVicar,^29^ noted that “commitment” is a multidimensional construct, which may also explain some of the variability noted in the statements. Among others, they posited that commitment can be conceptualized in either a behavioral or an attitudinal manner. Others have highlighted this multidimensionality.^17^

### Strengths and limitations

To our knowledge, this is the first large study that involves the commitment-to-change approach in palliative care. It involves over 2500 participants and 14,000 commitment statements and reflections. Participants provided their commitment statements, reflections and impact examples through free text, with no prompts to cue them and potentially introduce response biases.

Our study has several limitations. First, the reflections and implementation rates are self-reported by learners. This limitation is acknowledged by educators and researchers who use the commitment-to-change approach and those who study self-assessment of skills.^15,24,33,53^ Our study was not designed to independently confirm the response rates, verify the examples of impact provided by the learners or study how well the changes had been implemented. The commitment-to-change exercise in the LEAP courses is a low-stakes activity in that the issuance of continuing education certificates is not linked to whether learners implemented a change or not. Evidence of reflection is all that is required. There is therefore no apparent incentive to overstate, invent or embellish implementation and examples of impact or barriers experienced. Some learners may have been subject to social desirability (and overreported implementation or impact) or attribution (ascribed lack of implementation to external barriers when the reasons resided in them) biases.^54^ Reassuringly, there is evidence that reported changes submitted in commitment-to-change reflections are generally reliable.^15,24,25,31,33,55,56^

Second, the response rate for the 4-months commitment reflections was relatively low (23%). This may have introduced a self-selection bias in that learners who completed the reflections may have been more motivated to implement change and to report these than those who did not submit statements and reflections. However, our study may have also underestimated the impact of the courses as our commitment reflection form did not allow for learners to report new commitments, changes not linked to the commitments, or ongoing implementation efforts.^15,22,24,30^ We cannot assume that non-respondents did not implement changes into their practices, especially as there is evidence that completing a commitment statement, even without a follow-up reflection, is predictive of implementation.^24,25^

### What this research adds and implications for practice

These results are encouraging in that they reflect the course’s goals and learning objectives. This confirms course instructional design choices.^38^ Participants provided their commitment statements, reflections and impact examples through free text, with no prompts such as lists to cue them.

Strategies to increase compliance and implementation across the professions need to be incorporated.^29^ These include great emphasis during the course on the important role of the commitment-to-change activity, linking CTC to continuous quality improvement activity, and peer support.^20,29,57^

Commitments are also more likely to be implemented if they are clear, measurable, relatively easy to do, realistic, linked to timelines, within the scope of influence of the learner, and deemed important by the learner.^17,55,58,59^ In previous studies, learners who reported higher levels of confidence in a change were also more likely to implement it.^30^

In the context of using commitment-to-change to evaluate education programs, the importance of including other sources of evaluation information and not relying solely on commitment statements and reflections has previously been emphasized.^15,23,30^ Change in clinical practice is complex and the extent to which a single educational activity effects change is often not clear.

We believe that commitment-to-change reflections, consisting of hundreds of examples of impact on patients, services, and colleagues, even though self-reported, represent “higher levels” of evidence in evaluation frameworks such as Kirkpatrick’s and Moore frameworks.^60,61^ In the absence of pragmatic, easily available methods to collect reliable objective data at those higher patient and health care system levels, commitment-to-change provides some acceptable evidence of impact.^62,63^

### Future research

The study raises several questions that warrant future research. To what extent do self-reported commitment reflections mirror actual changes in practice? To what extent do non-responders make changes to their practices? Comparison groups may shed more light on the relative impact of the commitments-to-change.^32^ Are changes sustained on the longer term?

## Conclusions

This large study provides evidence, through commit-ment-to-change statements and reflections, that the LEAP Core courses directly benefit patients, health care professionals and primary care services, at least as self-reported by study participants. The study did not explore the full extent of the impact, or the quality of care provided, or system improvements made as a result of the changes. The benefits span a broad range of areas, including initiating a palliative care approach earlier across both cancer and non-cancer diseases, opioid use and symptom management, advance care planning and goals of care, communication and interprofessional collaboration.

The commitment-to-change approach is not a panacea and has its limitations, but it is an approach available to palliative care educators to enhance the application into practice of education interventions, foster reflective practice and provide useful data for curriculum development and evaluation.

## Presented previously in part at the following conferences

1. Annual International Conference of the North American Primary Research Group (NAPCRG). Chicago, United States of America. 11 Nov 2018.
2. 10th World Research Conference of the European Association of Palliative Care. Berne, Switzerland. 25 May 2018.
